# Microfibrillar-associated protein 4 as a predictive biomarker of treatment response in patients with chronic inflammatory diseases initiating biologics: secondary analyses based on the prospective BELIEVE cohort study

**DOI:** 10.1007/s00296-024-05744-9

**Published:** 2024-10-28

**Authors:** Bjørk K. Sofíudóttir, Heidi L. Munk, Robin Christensen, Sören Möller, Silja H. Overgaard, Grith L. Sorensen, Karen M. Møllegaard, Jessica Pingel, Anders B. Nexøe, Henning Glerup, Tanja Guldmann, Natalia Pedersen, Jens Frederik Dahlerup, Christian L. Hvas, Karina W. Andersen, Mohamad Jawhara, Ole Haagen Nielsen, Fredrik Olof Bergenheim, Anette Bygum, Jesper R. Davidsen, Signe Bek Sørensen, Jacob B. Brodersen, Jens Kjeldsen, Vibeke Andersen, Torkell Ellingsen

**Affiliations:** 1 Department of Rheumatology, Odense University Hospital, Odense, Denmark; 2 Research Unit of Rheumatology, Department of Clinical Research, University of Southern Denmark, Odense University Hospital, Odense, Denmark; 3 Section for Biostatistics and Evidence-Based Research, the Parker Institute, Bispebjerg and Frederiksberg Hospital, Copenhagen, Denmark; 4 Center for Rheumatology and Spine Diseases, Rigshospitalet, Glostrup, Denmark; 5 Cochrane Denmark & Centre for Evidence-Based Medicine Odense (CEBMO), Department of Clinical Research, University of Southern Denmark, Odense, Denmark; 6 OPEN – Open Patient Data Explorative Network, Odense University Hospital, Odense, Denmark; 7 Department of Clinical Research, University of Southern Denmark, Odense, Denmark; 8 Internal Medicine Research, Unit University Hospital of Southern Denmark, Aabenraa, Denmark; 9 Department of Regional Health Research, University of Southern, Odense, Denmark; 10 Department of Inflammation Research, Institute of Molecular Medicine, University of Southern Denmark, Odense, Denmark; 11 Department of Medical Gastroenterology, Odense University Hospital, Odense, Denmark; 12 University Research Clinic for Innovative Patient Pathways, Silkeborg Regional Hospital, Silkeborg, Denmark; 13 Department of Gastroenterology, Slagelse Hospital, Slagelse, Denmark; 14 Department of Hepatology and Gastroenterology, Aarhus University Hospital, Aarhus, Denmark; 15 Department of Surgery, University Hospital of Southern Denmark, Aabenraa, Denmark; 16 Department of Gastroenterology, Herlev Hospital, University of Copenhagen, Herlev, Denmark; 17 The Abdominal Center, Medical Section, Bispebjerg and Frederiksberg University Hospital, Copenhagen, Denmark; 18 Department of Respiratory Medicine, Odense University Hospital, Odense, Denmark; 19 Department of Gastroenterology, Esbjerg and Grindsted Hospital - University Hospital of Southern Denmark, Esbjerg, Denmark; 20 Research Unit of Medical Gastroenterology, Department of Clinical Research, University of Southern Denmark, Odense, Denmark

**Keywords:** Chronic inflammatory disease, Biologic treatment, Treatment response, MFAP4, Rheumatoid arthritis, Psoriatic arthritis, Axial spondyloarthritis, Psoriasis, Crohn’s disease, Ulcerative colitis

## Abstract

**Background:**

Currently, there are no reliable biomarkers for predicting treatment response in chronic inflammatory diseases (CIDs).

**Objective:**

To determine whether serum microfibrillar-associated protein 4 (MFAP4) levels can predict the treatment response to biological therapy in patients with CIDs.

**Methods:**

The BELIEVE study was originally designed as a prospective, multi-center cohort study of 233 patients with either rheumatoid arthritis, psoriatic arthritis, psoriasis, axial spondyloarthritis, Crohn’s disease, or ulcerative colitis, initiating treatment with a biologic agent (or switching to another). Clinical assessment and blood sample collection were performed at baseline and 14–16 weeks after treatment initiation. The primary analyses included participants with available blood samples at baseline; missing data were handled as non-responders. The patients were stratified into the upper tertile of serum MFAP4 (High MFAP4) versus a combined category of middle and lower tertiles (Other MFAP4). The primary outcome was the proportion of patients with clinical response to biologic therapy after 14–16 weeks.

**Results:**

211 patients were included in the primary analysis population. The mean age was 43.7 (SD: 14.8) years, and 120 (59%) were female. Positive treatment response was observed in 41 (59%) and 69 (49%) for High MFAP4 and Other MFAP4, respectively. When adjusting for pre-specified variables (CID, age, sex, smoking status, and BMI), the adjusted OR was 2.28 (95% CI: 1.07 to 4.85) for a positive treatment outcome in the High MFAP4 group.

**Conclusion:**

A high MFAP4 status before initiating biological treatment is associated with a positive clinical response, when adjusting for confounding factors.

**Supplementary Information:**

The online version contains supplementary material available at 10.1007/s00296-024-05744-9.

## Introduction

Chronic inflammatory diseases (CIDs), such as rheumatoid arthritis (RA), psoriatic arthritis (PsA), psoriasis (PSO), axial spondyloarthritis (AxSpA), Crohn’s disease (CD), and ulcerative colitis (UC) are recurring and lifelong diseases that have a negative impact on the quality of life of those affected [1–4]. Due to general population growth, an increased prevalence of CIDs is expected [1, 3, 5]. Drugs targeting the pro-inflammatory cytokine tumor necrosis factor-α (TNF-α), the so-called TNF-α inhibitors, as well as other biologics, are used to treat CIDs [6–9]. Biological agents are not without side effects [10], and about 40% of patients do not reach an adequate treatment response [11, 12]. Currently, no reliable biomarkers enable a valid prediction of treatment response [13, 14].

Microfibrillar-associated protein 4 (MFAP4) is an extracellular matrix (ECM) protein with high vascular expression and is also present in a circulatory form [15]. Increased serum MFAP4 is demonstrated to reflect disease-induced processes and has been correlated with the presence and severity of liver cirrhosis [16–18]. Increased circulatory MFAP4 is associated with increased 7-year mortality in patients with peripheral artery disease, as well as the presence of atrial fibrillation [19, 20]. In contrast, basal serum MFAP4 has relatively limited variation [21, 22]. Chronic inflammation can lead to persistent ECM remodelling with the potential release of MFAP4 to the circulation [15]. MFAP4 has been associated with the inflammatory and fibrotic process in chronic kidney disease and has been found to drive the eosinophilic inflammation in eksperimental asthma [23, 24]. Under the assumption that MFAP4 reflects the presence of inflammatory disease processes, that may be effectively treated by biologics, this study aimed to test whether high MFAP4 levels at baseline predict a positive treatment outcome in a broad selection of CID patients initiating or changing biologic therapy.

## Methods

This study is a secondary analysis of the Chronic Inflammatory Disease, Lifestyle and Treatment Response (BELIEVE) study, and methods are described in Overgaard et al. [25]. The study complies with the Declaration of Helsinki, and was approved by the Regional Committees on Health Research Ethics for Southern Denmark in 2016 (S-20160124) as well as the Danish Data Protection Agency (2008-58-035). Each participant signed an informed consent before inclusion to the study. The study was registered at Clinical.Trials.gov (NCT03173144), and a protocol was published before the initiation of the study [26]. A statistical analysis plan (SAP) was specified before initiating statistical analyses (Supplementary File). Findings are reported according to the STROBE statement [27].

### Patients

As previously described, this prospective study took place at nine Danish clinical centres [25, 26]. Patients with CIDs who were about to initiate treatment or change biologic agents were invited to participate. The included CIDs were: rheumatoid arthritis (RA), psoriatic arthritis (PsA), psoriasis (PSO), axial spondyloarthritis (AxSpA), Crohn’s disease (CD), and ulcerative colitis (UC). To be eligible, participants had to be minimum 18 years of age, and able to understand and sign an informed consent.

### Main outcome variable

Treatment response was based on specific disease activity measures for the individual CIDs. At the time of inclusion, all patients had active disease, according to individual CID guidelines, to initiate treatment or change to other biologic agents. Secondary outcomes included participant-reported changes in health-related quality of life as well as the physician’s global assessment and C-reactive protein (CRP) levels.

### Study factors

Groups were defined based on the serum MFAP4 levels, detected using the AlphaLISA technique, as described in Wulf-Johansson, H., et al. [22]. Serum samples were collected at baseline, before treatment initiation, and again 14–16 weeks following initiation of treatment. The primary outcome was the proportion of patients with a positive treatment response to biologic therapy (14–16 weeks) after treatment initiation as a group as well as individual CIDs. The specific criteria for positive treatment response varied across the CID conditions [26]: CD: clinical remission, defined as Harvey-Bradshaw Index of 4 or less; UCUC: clinical remission, defined as Mayo Clinic Score of 2 or less (with no individual sub-score of > 1); RA: clinical response, defined as at least a 20% improvement according to the criteria of the American College of Rheumatology (ACR20) [28]; AxSpa: clinical response, defined as at least a 20% improvement according to the Assessment of Spondyloarthritis International Society (ASAS20) [29]; PsA: clinical response, defined as at least a 20% improvement according to the criteria of ACR20; PSO: clinical response, defined as at least a 75% improvement in Psoriasis Area and Severity Index (PASI 75).

### Other variables

Secondary generic outcomes included changes from baseline to follow-up in measures of health-related quality of life (SF-12; the physical and mental component summaries (PCS and MCS, respectively), the short health scale consists of four components: symptom burden, functional status, disease-related burden and general wellbeing), C-reactive protein, and physician’s global assessment. Additionally, the proportion of patients continuing biologic treatment beyond the follow-up period served as a secondary outcome measure. Other secondary non-generic outcomes listed at clinical.trials.gov included changes from baseline to follow-up in disease scores (e.g., ∆HBI score, ∆Mayo Clinic score, ∆tender joint count, ∆swollen joint count, ∆PASI score, etc., see SAP in supplementary).

### Procedures

Participants’ blood samples were collected at inclusion prior to initiating therapy with biologics. Participants with missing blood samples at baseline were excluded from the analysis population. The examination program at inclusion also included health-related quality of life questionnaires, as well as clinical assessment. After 14–16 weeks of treatment, the participants were re-evaluated clinically for treatment response, blood samples were obtained, and health-related quality of life questionnaires were answered again. The inclusion period was between September 1st 2017 and March 31st 2020. Participants with available blood samples at baseline, were included in the intention to treat (ITT) population. They were stratified into two categories: the upper tertile of serum MFAP4 (High MFAP4) and a combined group joining the middle and lower tertiles of serum MFAP4 (Other MFAP4). The cut-off was pragmatically decided, assuming that a high MFAP4 level indicated high inflammatory disease activity, that could be blocked by biological treatment.

The study data were collected, pseudo-anonymized and managed using Research Electronic *Data* Capture database (REDCap) tools hosted at the Open Patient Data Explorative Network – Odense University Hospital storage facility (OPEN) [30]. Patients were included and evaluated after signed informed consent (baseline). Attending physicians assessed the disease activity of the participants and filled out CID-specific standardised data forms on disease activity at baseline and follow-up. These data forms were the Harvey Bradshaw Index (HBI, CD), the Mayo Clinic Score (UC), the Simple Clinical Colitis Activity Index (SCCAI, UC), the Simplified Disease Activity Index (SDAI, RA and PsA), the 46 joint count (RA), the Bath Ankylosing Spondylitis Metrology Index (BASMI, axSpA), the Psoriasis Area and Severity Index (PASI, PsO), the 66/68 joint count (PsA), as well as a global assessment (all). Qualified study personnel collected project-relevant clinical information from the patient’s medical records.

Patients reported on the generic quality of life measures [the Short Form Health Survey-12 (SF-12) and the Short Health Scale] as well as CID-specific quality of life measures [RA and PsA; Health Assessment Questionnaire Disability Index (HAQ-DI), PsO; Dermatology Life Quality Index (DLQI)]. Furthermore, patients reported disease activity scores relevant for AxSpA; Bath Ankylosing Spondylitis Functional Index (BASFI) and Bath Ankylosing Spondylitis Disease Activity Index (BASDAI). Smoking status was registered via electronic questionnaire at baseline and again at follow-up. More details are available in the statistical analysis plan (SAP) (see Supplementary File 1).

The study was approved by The Regional Committees on Health Research Ethics for Southern Denmark (S-20160124), and the processing of personal data was notified to and approved by the Region of Southern Denmark and listed in the internal record (18/13682) cf. Art 30 of the EU General DATA Protection Regulation. As reported by Overgaard et al. [25], two patient associations (the Danish Colitis-Crohn’s Association and the Danish Psoriasis Association) and three patient representatives diagnosed with RA were involved in developing recruitment plans as well as the design of the study and communication about the study to patient members.

### Statistical analysis

Statistical analyses followed the pre-specified statistical analysis plan (SAP) based on the analysis outlined in the original protocol and the first study published on this cohort [25, 26]. The treatment response was explored in relation to baseline MFAP4 levels, stratified into the upper tertile, 33.3% (High MFAP4) and lumping the middle and lower tertile, 66.67% (Other MFAP4). Standardised differences (StdD) were calculated to compare the distribution of baseline covariates between groups [31]: a standardised difference above 0.5 StdD-units was considered indicative of a potentially significant imbalance and difference between exposure groups at baseline. *P*-values were calculated using the Student’s t-test, Wilcoxon rank sum, or chi-squared test, as appropriate.

The independent contribution of the CIDs to the primary composite outcome was visualised in a forest plot: In the ITT population (using a simplistic non-responder imputation for missing outcome data), the ORs and 95% confidence interval (95% CI) of clinical responses in the groups within each CID were calculated and pooled the estimates using random-effects meta-analysis (STATA version 16.1, the “metan” package) as heterogeneity across CIDs was anticipated. Differences in the proportions of participants responding between groups were analysed using two different logistic regression analysis models: (i) in the “CID adjusted model,” an adjustment was made only for CID; whereas, in the adjusted model (ii) an adjustment for CID, sex, age, smoking status (ordinal scale: never, former, occasional, and current), as well as BMI category (ordinal scale: underweight, normal, overweight, and obese) was made, which were a priori considered potential confounding variables (See SAP).

The estimates for the continuous outcomes were reported as least squares (LS) means with standard errors. All *P*-values and 95% CIs were estimated and reported as two-sided; a *P* < 0.05 was considered potentially indicative of a statistically significant finding.

To assess the predictive value of serum MFAP4 levels on clinical response on a continuous scale (i.e., independent of arbitrary tertile thresholds), a receiver operating characteristic (ROC) curve analyses in an attempt to improve the threshold of serum MFAP4 levels best predicting a positive treatment response. The ROC curve was created by plotting the true positive rate (sensitivity) against the false positive rate (1 - specificity) at various threshold serum MFAP4 values. Each point on the curve represents a different threshold for classifying positive and negative instances. Youden’s index was used to find the optimal threshold for all CIDs and individual groups.

### Open data sharing

The datasets generated during and analysed during the current study are available from the corresponding author on reasonable request. Relevant authorities e.g., the Danish Data Protection Agency, must approve the data requestors, in adherence to GDPR regulations.

## Results

**Fig. 1:**
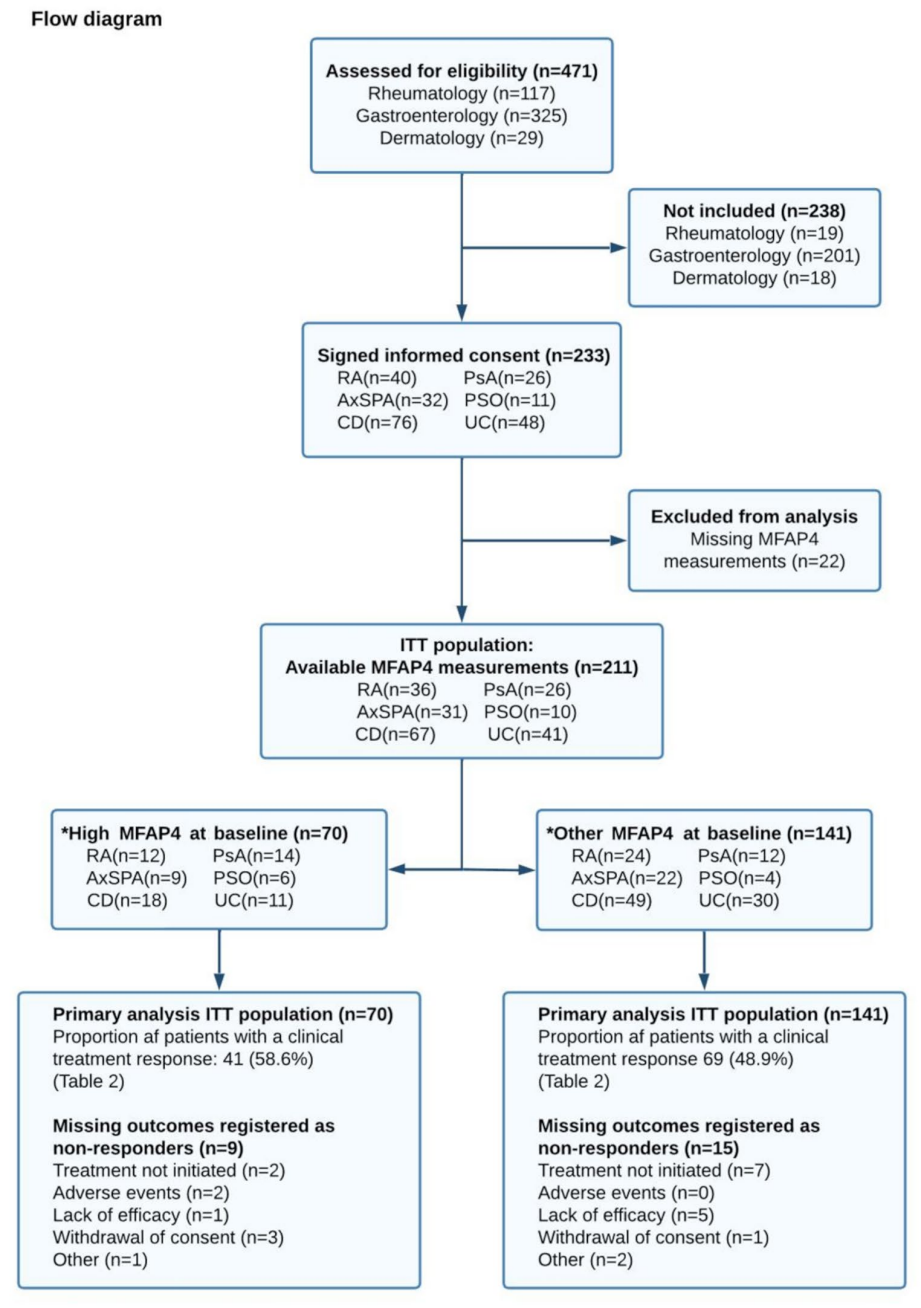
Flow Diagram. RA; Rheumatoid arthritis, PsA; Psoriatic arthritis, AxSPA; Axial Spondyloarthritis, PSO; Psoriasis, CD; Crohn’s disease, UC; Ulcerative Colitis, MFAP4; Microfibrillar-associated protein 4, ITT; Intention to treat. *MFAP4 levels are divided into tertiles; “High sMFAP4” is the upper tertile and “Other sMFAP4” is the medium and lower tertile

Of the 233 included participants, 211 had blood samples taken at baseline and were included in the primary analysis. Of these patients, 36 had RA, 26 had PsA, 31 had AxSPA, 10 had PSO, 67 had CD, and 41 had UC (Fig. 1). The mean age was 43.7 (standardised difference (SD): 14.8) years and 120 (59%) were female. Of the 211 patients in the primary analysis, 41 participants had previously been treated with biologics or were changing from one biologic treatment to another; of these, 23 (56%) had CD, 8 (20%) had UC, 7(17%) had PSO, 2 (5%) had PsA, and 1 (2%) had AxSPA.

Baseline characteristics and descriptive statistics off the participants, stratified into High MFAP4 and Other MFAP4, are listed in Table 1. The mean age of the High MFAP4 was 50.5 (SD: 14.1), and in the Other MFAP4, the mean age was 40.4 (SD: 14.0). Age was the only variable with a significant difference between the groups, with a standardised difference (StdD) of > 0.5. All other variables were relatively balanced between the groups.

**Table 1:** Baseline characteristics

Characteristic	Total *n* = 211	High MFAP4 *n* = 70	Other MFAP4 *n* = 141	StdD*	*P*-value
Age (years), mean (SD)	43.7 (14.8)	50.5 (14.1)	40.4 (14.0)	0.7*	< 0.001*
Female, *n* (%)	120 (59%)	42 (64%)	78 (57%)	0.1	0.446
BMI (kg/m^2^), mean (SD)	27.2 (6.1)	28.1 (6.9)	26.8 (5.6)	0.2	0.175
Disease duration (years)	5.0 (2.0; 12.0)	7.0 (2.5; 13.0)	4.0 (1.0; 10.0)	0.4	0.013*
Smoking status, *n* (%):					0.584
Non-smoker	81 (43%)	28 (47%)	53 (41%)	0.1	
Former smoker	69 (37%)	21 (36%)	48 (38%)	0.0	
Occasionally	4 (2%)	2 (3%)	2 (2%)	0.1	
Daily	33 (18%)	8 (14%)	25 (20%)	-0.2	
CID diagnosis, *n* (%):					0.072
Rheumatoid arthritis	36 (17%)	12 (17%)	24 (17%)	0.0	
Psoriatic arthritis	26 (12%)	14 (20%)	12 (9%)	0.3	
Axial spondylarthritis	31 (15%)	9 (13%)	22 (16%)	-0.1	
Psoriasis	10 (5%)	6 (9%)	4 (3%)	0.2	
Crohn’s disease	67 (32%)	18 (26%)	49 (35%)	-0.2	
Ulcerative colitis	41 (19%)	11 (16%)	30 (21%)	-0.1	
Naïve to biological treatment, *n* (%):	166 (80%)	54 (78%)	112 (81%)	-0.1	0.712
Medication at inclusion, *n* (%)					
None	17 (8%)	4 (6%)	13 (9%)	-0.1	0.435
NSAID, daily use	21 (12%)	8 (14%)	13 (11%)	0.1	0.618
Corticosteroids	69 (33%)	22 (31%)	47 (33%)	-0.0	0.876
Immunomodulators	75 (36%)	29 (41%)	46 (33%)	0.2	0.224
5-ASA/SASP	54 (26%)	16 (23%)	38 (27%)	-0.1	0.616
Leflunomide	7 (3%)	4 (6%)	3 (2%)	0.2	0.224
Hydroxycloroquine	19 (9%)	6 (9%)	13 (9%)	-0.0	1.000
Antibiotics	4 (2%)	0 (0%)	4 (3%)	-0.2	0.304
Secondary outcome measures Scales (0-100):					
SF-12 PCS	41.8 (38.6; 45.7)	41.3 (37.5; 44.5)	41.9 (38.9; 46.1)	-0.3	0.113
SF-12 MCS	47.0 (41.6; 52.2)	49.7 (44.3; 55.1)	46.5 (41.1; 51.8)	0.5*	0.008*
SHS-Symptom burden	63.5 (50.0; 75.0)	59.5 (50.0; 74.0)	65.0 (51.0; 75.5)	-0.1	0.392
SHS-Functional status	62.0 (35.0; 77.0)	51.5 (30.0; 72.0)	64.5 (39.0; 79.0)	-0.3	0.081
SHS-Disease-related burden	67.0 (46.0; 82.0)	62.0 (40.0; 78.0)	71.0 (46.5; 84.0)	-0.2	0.100
SHS-General well-being	54.0 (35.0; 72.0)	50.0 (30.0; 66.0)	55.5 (40.0; 72.5)	-0.1	0.260
Phys. Global assessment	50.0 (31.0; 68.0)	40.0 (30.0; 60.0)	52.0 (35.0; 71.0)	-0.4	0.023*
CRP, mg/L	3.9 (1.7; 12.0)	3.6 (2.1; 14.0)	3.9 (1.6; 12.0)	-0.2	0.668
Exploratory outcome measure:					
MFAP4 (U/mL)	27.2 (20.2; 34.7)	40.9 (34.7; 46.5)	23.1 (17.1; 27.2)	N/A	N/A

Numbers are median (IQR), unless stated otherwise, *=Moderate effect size, **=Large effect size. *P*-value* <0.05. NSAID = Non-steroidal anti-inflammatory drugs, Immunomodulators = Methotrexate, Azathioprine, or 6-mercaptopurine, 5ASA/SASP = 5-aminosalicylic acid/salazosulfapyridin. Abbreviations: ITT = Intention to treat, n = number, StdD = Standardized difference (Cohen’s d), BMI = Body mass index, SF-12 = the Short Form Health Survey-12, PCS = physical component summaries, MCS = mental component summaries, SHS = Short Health Scale

**Fig. 2:**
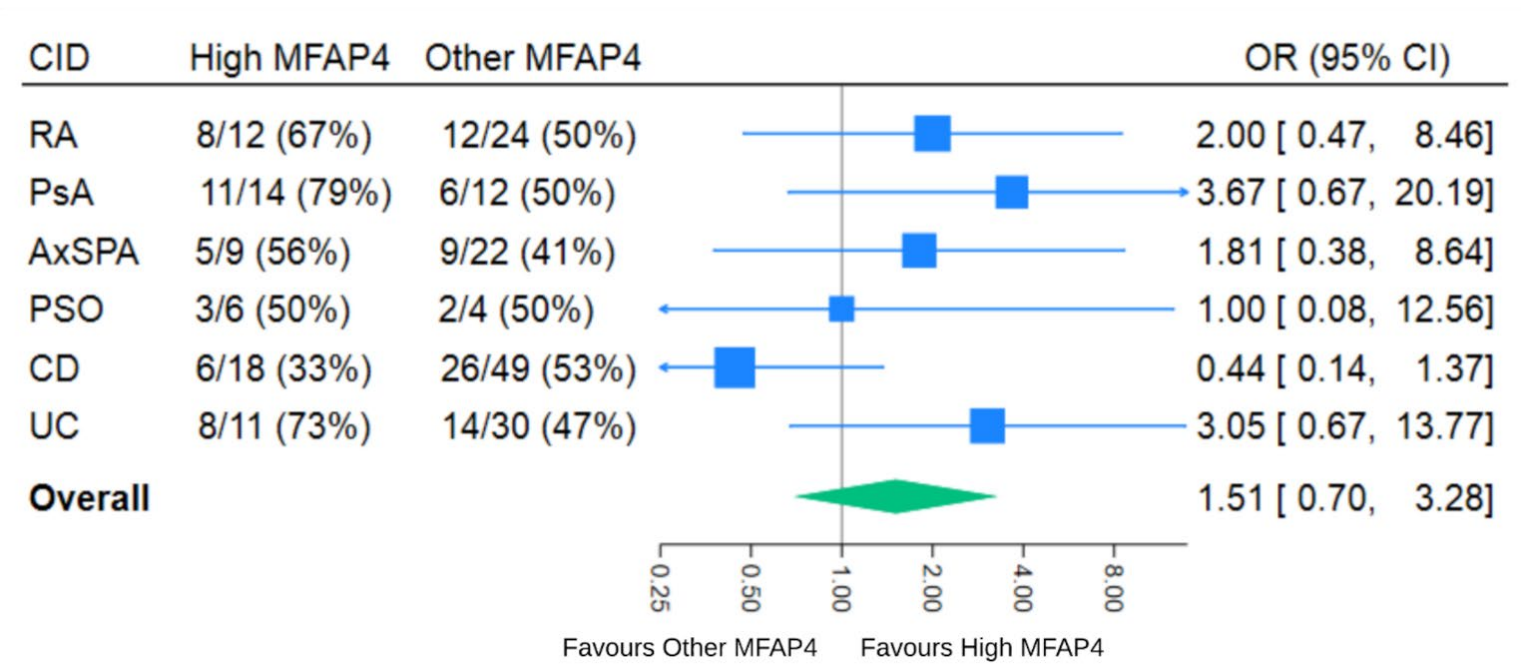
Forest plot; Effect of MFAP4 profile on treatment response. Values are presented as responder/all and % **Explanation**: CID = Chronic Inflammatory Disease, OR = Odds ratio, CI = Confidence interval, High MFAP_4_ =  the upper 33.3% of the study sample based on serum MFAP_4_ measurements, Other MFAP_4_ = the lower 66.7% of the study sample based on serum MFAP_4_ measurements

**Fig. 3:**
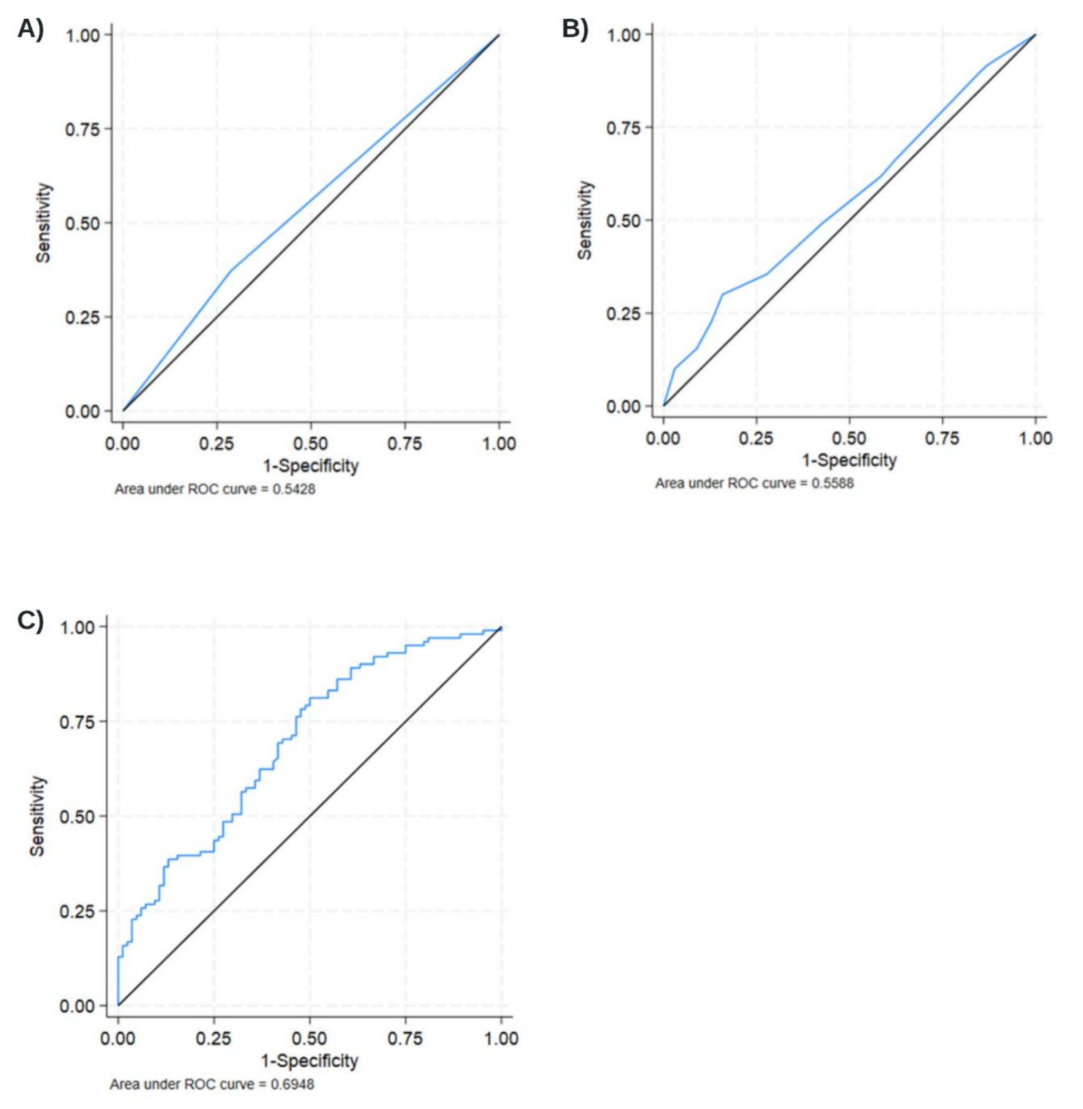
Receiver operator curve (ROC) for levels of MFAP4 in predicting treatment response in all CIDs. (**A**) is not adjusted (Crude), (**B**) is CID adjusted, and (**C**) is the fully adjusted model

A total of 110 (52%) of the 211 participants in the ITT population had a positive clinical response to biologics. In the High MFAP4 group, 41 (59%) had a clinical response, while 69(49%) in the Other MFAP4 group had a clinical response (Table 2). There was no difference between the groups’ positive treatment responses in the primary CID-adjusted analysis (OR 1.39, 95% CI: 0.77 to 2.53), for High MFAP4, but when also adjusting for age, sex, smoking status, and BMI, the resulting OR was 2.28 (95% CI: 1.07 to 4.85), in favour of High MFAP4. When looking at the subgroups, most CIDs seem to favour the High MFAP4 group for a positive treatment response. As an exception, CD seemed to favour the Other MFAP4 group. The OR for a positive treatment response in the two MFAP4 groups in the individual CIDs, is presented as a forest plot (Fig. 2). In the secondary outcome measures, High MFAP4 was positively correlated with SF-12 PCS, adjusted LS mean of 2.30 (95% CI 0.13 to 4.47), and a negative correlation to SF-12MCS, adjusted LS mean of -4.05 (95% CI: -7.00 to -1.10). No other secondary outcome variables differed significantly between the groups. ROC curves were calculated for treatment response for various MFAP4 levels for the crude model (Fig. 3). The optimal MFAP4 level cut point (post hoc), for predicting treatment response was interpreted based on the Youden’s index. The empirical data-driven optimal cutpoint was 34.6 U/mL. Sensitivity at cutpoint (sens.): 0.31, Specificity at cutpoint (spec.): 0.80, Area under ROC curve at cut point: 0.56.

**Table 2:** Primary and secondary outcome measures comparing high and other MFAP4 groups

Outcome	High MFAP4 *n* = 70	Other MFAP4 *n* = 141	CID adjusted^1^		Adjusted^2^	
			Contrast (95%CI)	*P*-value*	Contrast (95%CI)	*P*-value*
Primary outcome (composite)						
Positive treatment response, *n* (%)	41 (59%)	69 (49%)	1.39 (0.77 to 2.53)	0.274	2.28 (1.07 to 4.85)	0.033*
Sub-components, *n* (%)						
Rheumatoid arthritis, ACR20	8 (67%)	12 (50%)	2.00 (0.47 to 8.46)		4.83 (0,27 to 85,75)	
Psoriatic arthritis, ACR20	11 (79%)	6 (50%)	3.67 (0.67 to 20.19)		6.07 (0.36 to 101.63)	
Axial spondyloarthritis, ASAS20	5 (56%)	9 (41%)	1.81 (0.38 to 8.64)		16.61 (0.55 to 501.47)	
Psoriasis, PASI75	3 (50%)	2 (50%)	1.00 (0.08 to 12.56)		N/A	
Crohn’s disease, HBI ≤ 4	6 (33%)	26 (53%)	0.44 (0.14 to 1.37)		0.45 (0.10 to 2.09)	
Ulcerative colitis, Mayo ≤ 2	8 (73%)	14 (47%)	3.05 (0.67 to 13.77)		8.89 (0.86 to 91.57)	
Key secondary outcomes Health-related quality of life (0-100):						
∆ SF-12 PCS	-0.32	-1.79	1.43 (-0.61 to 3.48)	0.169	2.30 (0.13 to 4.47)	0.038*
∆ SF-12 MCS	-0.97	2.21	-3.62 (-6.40 to -0.85)	0.011*	-4.05 (-7.00 to -1.10)	0.008*
∆ SHS-Symptom burden	25.27	23.39	0.46 (-8.63 to 9.55)	0.920	3.10 (-6.49 to 12.69)	0.524
∆ SHS-Functional status	21.88	21.64	-1.06 (-10.80 to 8.72)	0.831	0.75 (-9.30 to 10.80)	0.883
∆ SHS-Disease-related burden	22.02	21.13	-1.57 (-12.1 to 9.01)	0.770	0.40 (-11.1 to 11.94)	0.945
∆ SHS-General well-being	18.31	17.36	-0.89 (-10.5 to 8.73)	0.885	0.70 (-9.47 to 10.86)	0.892
∆ SHS- Physicians global assessment	35.07	36.07	0.09 (-9.74 to 9.93)	0.985	-0.71 (-11.60 to 10.22)	0.898
∆ CRP (mg/l)	3.09	1.08	1.50 (-9.84 to 12.84)	0.794	2.08 (-10.90 to 15.12)	0.752
∆ MFAP4 (U/ml)	-7.37	-6.38	-0.85 (-5.02 to 3.33)	0.690	-0.35 (-5.33 to 4.62)	0.888
Safety/harms, *n* (%)						
Continuation of treatment, no. (%)	56 (80%)	112 (79%)				
Withdrawals	3 (4%)	1 (1%)				
Discontinuation due to adverse events	5 (7%)	5 (4%)				
Serious adverse events (SAEs)	1 (1%)	3 (2%)				
Deaths	0 (0%)	0 (0%)				

Contrast: Results are OR for proportions and least squared means for continuous values. 1 = Adjusted for CID, 2 = Adjusted for age, sex, smoking status, and BMI. Abbreviations: n = number, ACR20 = the American College of Rheumatology ≥ 20% improvement, ASAS20 = the Assessment of Spondyloarthritis International Society ≥ 20% improvement, PASI 75 = the Psoriasis Area and Severity Index ≥ 75% improvement, Mayo = Mayo Clinic Score, HBI = Harvey Bradshaw Index, SF-12 = the Short Form Health Survey-12, PCS = physical component summaries, MCS = mental component summaries, SHS = Short Health Scale

Exploratory analysis of baseline MFAP4 measures showed a positive correlation between high MFAP4 and age as well as BMI, but not with current smoking (Table 3). Further, the optimal MFAP4 cut-off levels for the individual CIDs were calculated: RA had a cut-point of 34.6 U/ml, with a sensitivity 35% and specificity of 88%, PsA had a cut-point of 33.1 U/ml (sensitivity of 65% and specificity of 89%), AxSPA had a cut point at 29.8 U/ml (sensitivity of 50% and specificity of 76%), PSO had a cut point at 33.3 U/ml (with a sensitivity of 60% and specificity of 60%), CD had a cut point at 11.0 U/ml (sensitivity of 100% and specificity of 6%), UC had a cut point of 31.4 U/ml (sensitivity of 41% and specificity of 84%). ROC curves of all the individual CIDs are presented in Supplementary Figure S1.

**Table 3:** Exploratory analyses. Descriptive analysis of MFAP4 levels in relation to positive treatment response, clinical characteristics and change in MFAP4 (∆ MFAP4: after - baseline) based on Spearman’s Rank-Order correlation

	Baseline MFAP4	∆ MFAP4
Positive treatment response	*r* = 0.05 *p* = 0.486	*r*= -0.03 *p* = 0.681
Age	*r* = 0.37 *p* = 0.000*	*r* = 0.07 *p* = 0.342
Female	*r* = 0.15 *p* = 0.033	*r* =-0.03 *p* = 0.739
BMI	*r* = 0.16 *p* = 0.024*	*r* =-0.05 *p* = 0.514
Current smoker	*r* =-0.10 *p* = 0.183	*r* =-0.00 *p* = 0.996
Baseline CRP (mg/L)	*r* =-0.04 *p* = 0.637	*r* =-0.07 *p* = 0.397
*Change from baseline*:		
∆ SF-12 PCS (0-100)	*r* = 0.10 *p* = 0.238	*r* =-0.14 *p* = 0.117
∆ SF-12 MCS (0-100)	*r* =-0.07 *p* = 0.421	*r* = 0.10 *p* = 0.265
∆ SHS-Symptom burden (0-100)	*r* = 0.04 *p* = 0.661	*r* = 0.03 *p* = 0.726
∆ SHS-Functional status (0-100)	*r* = 0.04 *p* = 0.648	*r* = 0.05 *p* = 0.592
∆ SHS-Disease-related burden (0-100)	*r* =-0.01 *p* = 0.869	*r* = 0.00 *p* = 0.991
∆ SHS-being being well-being (0-100)	*r* = 0.03 *p* = 0.757	*r* = 0.06 *p* = 0.466
∆ Physicians global assessment (0–100 mm VAS)	*r* =-0.03 *p* = 0.755	*r* =-0.06 *p* = 0.565
∆ CRP (mg/L)	*r* = -0.14 *p* = 0.106	*r* =-0.05 *p* = 0.567

Abbreviations: r = Spearman’s rho, *p* = *P*-value, BMI = Body mass index, SF-12 = the Short Form Health Survey-12, PCS = physical component summaries, MCS = mental component summaries, SHS = Short Health Scale

Of the participants in the primary analysis, 83% (Range 75–92%) continued biological treatment, the lowest number of individuals continuing the treatment was among those with RA (75%) and the highest was among those with CD (92%) (Supplementary Table S1). Regarding of type of biological treatments used in this study, a total of 205 registrations were available. The majority received TNFi: *n* = 177, followed by α4β7 integrin inhibitor: *n* = 21, IL17i: *n* = 6 and IL12/IL23i: *n* = 1. There were 41 participants who had tried biological therapy before entering the study. Of these, 34% had a positive treatment response versus 58% in the bio-naïve group. There is a descriptive table (Table S2) available in supplementary, with the number of biologics prior to entering the study, and treatment response to biologics.

## Discussion

In our primary analysis, High MFAP4 was significantly associated with treatment response when adjusting for CID, age, sex, smoking status and BMI. This supports the theory of MFAP4 as a biomarker of active inflammation affected by biologics. When considering the CID subgroups except for Crohn´s disease, being in a High MFAP4 (having a high serum MFAP4 level) predicts a positive treatment response to biologic therapy; however, the subgroups were underpowered for individual analysis, resulting in wide CI’s. Being in the Other MFAP4 group predicted a positive treatment response in Crohn’s disease. This might indicate different inflammatory mechanisms in CD versus the other CIDs in this cohort. However, 23 (34%) of the CD patients were treated with biologics before entering this study, and it is unsure, whether that may have affected circulating MFAP4 levels. The present results support that MFAP4 has the potential to predict treatment response in CIDs, with a positive correlation to RA, PsA, AxSpA, and UC and seemingly a negative correlation to treatment response in CD. Thereby, MFAP4 has the potential to support personalised medicine by identifying the patients who are most likely to benefit from biological treatment when adjusting for CID, age, sex, smoking status, and BMI. The CID adjusted analysis was without significant difference, in relation to treatment response in the High MFAP4 and Other MFAP4 group. Age, sex, BMI and smoking are known to influence circulating MFAP4 levels [32–34]. Our study supports, that when applying MFAP4 as a biomarker of disease induced processes, these variables have to be taken into account.

In the secondary outcome measures, the SF12 measures had significant differences, where those with High MFAP4 seemed to have an improvement in SF12-PCS in the adjusted model. Still, the correlation was negative in SP12-MCS in both the crude and the adjusted model (Table 2). However, the observed changes have an absolute difference of 2.30 and − 4.05 for PCS and MCS, respectively, on a scale that has a range of 0-100. A study on minimally important changes in different outcome measures in RA determined that the minimally important change ranged from 2.6 to 4.4 and 2.2 to 4.7 for PCS and MCS, respectively [35]. The difference observed in PCS in this study is below the minimally important range and, therefore, not expected to be of clinical value. In contrast, MCS is within the minimally important range and may be of some importance. There was no other statistically significant difference among the secondary outcomes. Age was the only significant difference between the High MFAP4 and the Other MFAP4, consistent with the findings of other studies, indicating that MFAP4 increases with age [21, 34].

Previous studies have found that MFAP4 may be a marker of fibrotic liver disease, active inflammation in the lungs and blood vessels, and skin disease [15]. In our subgroup analysis, PsA seemed to favour high MFAP4, a condition involving both joints and the skin. This could be attributed to a relatively high release of MFAP4 from cell turnover from active skin and joint inflammation. The PSO group was too small to give any clear signal in the subgroup analysis, and 7 (70%) in the PSO group were not naïve to biologics, which may influence circulating MFAP4 levels. Other studies have observed underlying cardiovascular disease (CVD) and airway diseases (i.e., asthma and COPD) as potential causes of High MFAP4 [24, 32, 36–39]. Further, MFAP4 levels are increased in liver and renal disease, however, in renal disease, this may be to underlying CVD [33, 40, 41]. This indicates that CVD and pulmonary disease, and possibly other comorbidities, are potential confounders. Unfortunately, comorbidities could not be adjusted for in this study, as this information was not available. A strength of this study is the inclusion of CID patients about to initiate biological therapy or change to another type of biological therapy, making it possible to test for predictive markers of treatment outcome. Further, the ROC curve cut-off at 34.6 was just below the 95% CI of the high MFAP4 group (95% CI 34.7; 46.5), indicating that the upper tertile of MFAP4 was close to the optimal cut point. However, one limitation is the relatively small number of individual CID diagnoses, so the sub-group analysis did not carry enough statistical strength on the individual disease levels. Another limitation is that no data on CVD and pulmonary disease in the participants were available in this study, which may have influenced MFAP4 levels.

## Conclusions

When adjusting for confounders for MFAP4 levels, (CID, age, sex, smoking status and BMI), High MFAP4 showed a positive association with treatment outcome in all CIDs except Crohn’s disease, enabling High MFAP4 as a potential biomarker for biological treatment response. Future research should include measurements of MFAP4 in larger cohorts of the individual CIDs: RA, PsA, AxSpA, PSO, CD and UC initiating biological therapy.

## Electronic supplementary material

Below is the link to the electronic supplementary material.

Supplementary Material 1

## Declarations

### Disclaimer

This study is part of a PhD thesis, and some of the main findings of this study have been presented in the PhD thesis. The thesis can be accessed here: Bjork.K.Sofiudottir_rsyd.dk.pdf_reduced.pdf (sdu.dk).
