# Supplementary material for: Microfibrillar-associated protein 4 as a predictive biomarker of treatment response in patients with chronic inflammatory diseases initiating biologics: secondary analyses based on the prospective BELIEVE cohort study
Source: Rheumatol Int. 2024 Oct 28;44(12):2935–47. doi: 10.1007/s00296-024-05744-9 (PMC11618207; doi:10.1007/s00296-024-05744-9)
Supplement: Supplementary file 1 — Supplementary Material 1 [file 296_2024_5744_MOESM1_ESM.docx]

| **Appendix**  **Appendix table S1:** Changes from baseline in all outcome measures stratified by CID. Values are medians (IQR), unless otherwise stated | | | | | | | |
| --- | --- | --- | --- | --- | --- | --- | --- |
|  | RA | PsA | axSpA | PsO | CD | UC | All CIDs |
| ∆ SF-12 PCS (0-100) | -1.5  (-5.5; 2.4) | -0.5  (-4.0; 1.1) | -3.1  (-5.2; 2.8) | -0.9  (-2.6; 6.2) | -1.5  (-4.5; 0.9) | 0.9  (-1.4; 2.2) | -1.1  (-4.2; 2.2) |
| ∆ SF-12 MCS (0-100) | 0.6  (-4.2; 6.1) | 1.7  (-4.9; 6.1) | 2.6  (-5.4; 6.1) | -1.9  (-4.4; 11.1) | 0.7  (-2.7; 5.6) | -0.4  (-3.9; 3.5) | 0.6  (-4.0; 5.9) |
|  |  |  |  |  |  |  |  |
| **∆ Short health scale (0-100**): |  |  |  |  |  |  |  |
| ∆ Symptom burden | 15.0  (4.0; 41.0) | 27.0  (11.0; 47.0) | 24.0  (9.0; 46.0) | 49.5  (30.0; 56.0) | 17.0  (4.0; 29.0) | 30.0  (18.0; 37.0) | 21.0  (8.0; 42.0) |
| ∆ Functional status | 14.0  (0.0; 36.0) | 23.0  (7.0; 47.0) | 32.0  (6.0; 49.0) | 21.5  (11.0; 61.0) | 17.0  (5.0; 31.0) | 15.0  (8.0; 34.0) | 17.0  (5.0; 39.5) |
| ∆ Disease-related burden | 11.0  (-3.0; 37.0) | 29.5  (13.0; 49.0) | 25.0  (9.0; 41.0) | 41.5  (0.0; 65.0) | 15.0  (2.0; 39.0) | 19.0  (4.0; 34.0) | 19.0  (2.0; 38.5) |
| ∆ General well-being | 13.0  (0.0; 34.0) | 24.0  (14.0; 39.0) | 21.0  (0.0; 40.0) | 3.5  (-8.0; 70.0) | 12.0  (1.0; 33.0) | 15.0  (8.0; 25.0) | 16.0  (2.0; 34.5) |
|  |  |  |  |  |  |  |  |
| ∆ MFAP4 (U/mL) | 7.6  (1.2; 17.2) | 4.3  (-0.1; 12.9) | 3.1  (1.0; 7.0) | 0.8  (-1.1; 13.8) | 1.1  (-2.7; 6.8) | 6.9  (1.1; 12.9) | 3.7  (-0.0; 10.8) |
| ∆ CRP (mg/L) | 0.9  (0.1; 7.0) | 1.9  (0.2; 3.1) | 2.3  (0.0; 21.5) | 0.8  (0.2; 4.0) | 0.0  (-0.3; 2.2) | 0.0  (-2.1; 3.3) | 0.7  (0.0; 6.0) |
| ∆ PGA (0-100) | 37.5  (20.0; 45.0) | 30.0  (23.0; 35.0) | 45.0  (22.0; 64.0) | N/A | 38.0  (2.0; 60.5) | 59.0  (48.5; 64.0) | 35.0  (21.5; 55.0) |
| Continuation of treatment, no. (%) | 27 (75) | 20 (80) | 25 (83) | 9 (90) | 56 (92) | 31 (78) | 168 (83) |
| Figure S1 abbreviations: SF-12=the Short Form Health Survey-12, PCS= physical component summaries, MCS=mental component summaries, MFAP4=Microfibrillar-associated protein 4, CRP=C-reactive protein, PGA= Physicians global assessment | | | | | | | |

**Figure S1**: ROC-curves for the individual CIDs


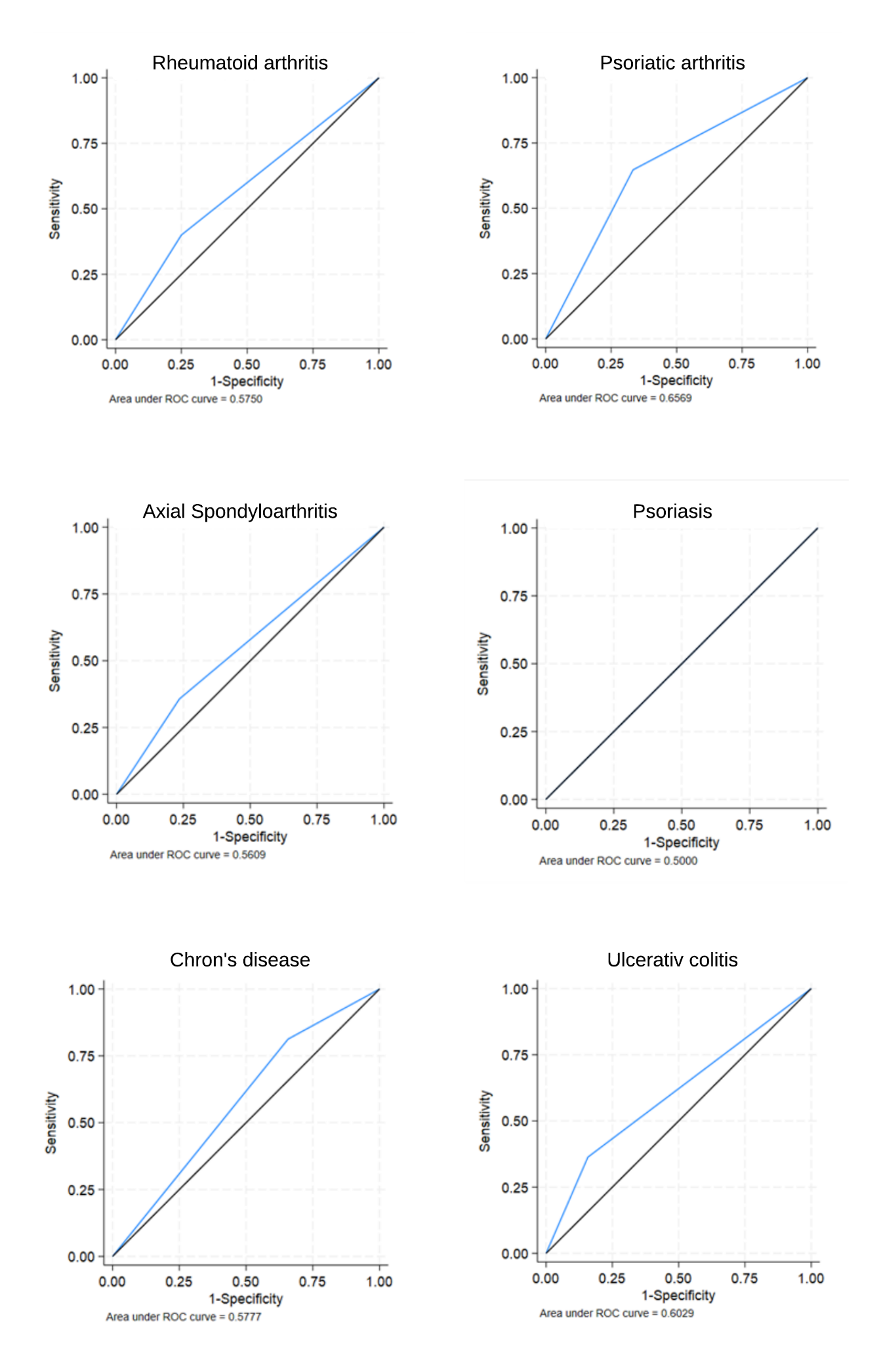


**Table S2:** Number of previous biological medications and clinical treatment response. Results are number and percentage (%).

|  | Number of biological medications used before entering the study | | | | | | | Total |
| --- | --- | --- | --- | --- | --- | --- | --- | --- |
|  | 0 | | 1 | | 2 | | ≥3 |  |
| Clinical Responder | 96 (58) | | 7 (27) | | 6 (50) | | 1 (33) | 110 |
| Clinical non-responder | 70 (42) | | 19 (73) | | 6 (50) | | 1 (33) | 97 |
| **Total** | **166** | **26** | | **12** | | **3** | | **207** |
